# Progression of Selected Parameters of the Clinical Profile of Patients with Periodontitis Using Kohonen’s Self-Organizing Maps

**DOI:** 10.3390/jpm13020346

**Published:** 2023-02-16

**Authors:** Agata Ossowska, Aida Kusiak, Dariusz Świetlik

**Affiliations:** 1 Department of Periodontology and Oral Mucosa Diseases, Medical University of Gdansk, 80-208 Gdańsk, Poland; 2 Division of Biostatistics and Neural Networks, Medical University of Gdansk, 80-211 Gdańsk, Poland

**Keywords:** periodontitis, diagnosis, computer simulation, artificial neural networks, self-organizing maps

## Abstract

(1) Background: Periodontitis is an inflammatory condition that affects the tissues surrounding the tooth and causes clinical attachment loss, which is the loss of periodontal attachment (CAL). Periodontitis can advance in various ways, with some patients experiencing severe periodontitis in a short period of time while others may experience mild periodontitis for the rest of their lives. In this study, we have used an alternative methodology to conventional statistics, self-organizing maps (SOM), to group the clinical profiles of patients with periodontitis. (2) Methods: To predict the periodontitis progression and to choose the best treatment plan, we can use artificial intelligence, more precisely Kohonen’s self-organizing maps (SOM). In this study, 110 patients, both genders, between the ages of 30 and 60, were included in this retrospective analysis. (3) Results: To discover the pattern of patients according to the periodontitis grade and stage, we grouped the neurons together to form three clusters: Group 1 was made up of neurons 12 and 16 that represented a percentage of slow progression of almost 75%; Group 2 was made up of neurons 3, 4, 6, 7, 11, and 14 in which the percentage of moderate progression was almost 65%; and Group 3 was made up of neurons 1, 2, 5, 8, 9, 10, 13, and 15 that represented a percentage of rapid progression of almost 60%. There were statistically significant differences in the approximate plaque index (API), and bleeding on probing (BoP) versus groups (*p* < 0.0001). The post-hoc tests showed that API, BoP, pocket depth (PD), and CAL values were significantly lower in Group 1 relative to Group 2 (*p* < 0.05) and Group 3 (*p* < 0.05). A detailed statistical analysis showed that the PD value was significantly lower in Group 1 relative to Group 2 (*p* = 0.0001). Furthermore, the PD was significantly higher in Group 3 relative to Group 2 (*p* = 0.0068). There was a statistically significant CAL difference between Group 1 relative to Group 2 (*p* = 0.0370). (4) Conclusions: Self-organizing maps, in contrast to conventional statistics, allow us to view the issue of periodontitis advancement by illuminating how the variables are organized in one or the other of the various suppositions.

## 1. Introduction

Periodontitis is an inflammatory condition that affects the tissues surrounding the tooth and causes clinical attachment loss, which is the loss of periodontal attachment (CAL). Gingival tissue, alveolar bone, cementum, and periodontal ligaments make up the tooth’s supporting structure (periodontium). Gingivitis, an infection of the gingiva mainly carried on by tooth plaque, is the most common and mildest form of periodontitis. The gingiva alterations begin if the microbial biofilm is not properly removed within a few days or weeks. The patient frequently experiences halitosis, hemorrhage, edema, and redness of the gingiva [1,2,3,4]. Apart from bleeding, pain, and enlargement, erythema, edema, and bleeding are typical clinical symptoms of plaque-induced gingivitis [5,6]. Biological changes, diabetes, leukemia, smoking, malnutrition, and hormonal changes are all potential factors that can influence plaque-induced gingivitis. Hormonal alterations, hyperglycemia, leukemia, smoking, malnutrition, prominent subgingival restoration margins, and hyposalivation are potential modifying variables of plaque-induced gingivitis [7,8,9]. Periodontitis and increasing attachment loss are thought to require gingivitis as a prerequisite. Not everyone who has gingivitis will progress to periodontitis because this process is significantly correlated with the patient’s immune-inflammatory response [10,11].

Periodontitis can advance in various ways, with some patients experiencing severe periodontitis in a short period of time while others may experience mild periodontitis for the rest of their lives. Additionally, the evolution of periodontitis differs depending on the patient and is less predictable in certain cases than in others. In addition to weight, genetics, physical activity, or nutrition, well-known risk factors for accelerated bone loss include nicotine dependence and poorly managed diabetes. Furthermore, nicotinism is a major risk factor for the changes in oral mucosa such as leukoplakia [12]. The age of the patient is taken into consideration when the doctor evaluates the stage of periodontitis, which is an indirect technique to measure each patient’s vulnerability to periodontitis. The measurement of bone loss on radiograms expressed as a percentage of tooth length and divided by the patient’s age is a popular method of assessing bone loss in daily practice. In recent years, dentists assessed the typical clinical attachment loss for the patient’s age by comparing clinical attachment loss (CAL) with age. The UNC 15 standard probe can be used to make this measurement [13,14].

Artificial intelligence (AI) is gaining importance in the fields of medicine and dentistry nowadays. It can be beneficial in a variety of areas where helping humans is possible. It can be useful in many situations where new technologies might benefit and help people. In the above study, Kohonen’s self-organizing maps (SOM) were used. Artificially intelligent systems have the ability to remotely conduct quantitative calculations and can recognize aspects in clinical photographs that human specialists hardly ever discover. Deep learning algorithms are frequently utilized in picture prediction and diagnosis due to their advantages in terms of speed, accuracy, and reproducibility [15,16,17].

The aim of this study was to assess the progression and grade of periodontitis with the usage of given data and with the help of the self-organizing model. In this study, we have used an alternative methodology to conventional statistics, self-organizing maps (SOM), to group the clinical profiles of patients with periodontitis. Using this technique, we will be able to divide the study participants into a specific number of neurons. The value of each research variable relating to each of those neurons will be determined using the SOM algorithm. This allows for the simultaneous visualization of the values of each study variable in each group of patients contained in a neuron. With the use of this grouping technique, we can see how each variable affects the various patient groups and identify behavioral patterns that are related to a particular variable, in this case, the requirement to carry out a fenestration.

## 2. Materials and Methods

### 2.1. Patients’ Population

This was a retrospective study, and the database consisted of 110 patients; both genders aged 30 to 60 were included. The selection of the patients was performed in 2022 in the Department of Periodontology and Oral Mucosa Diseases, Medical University of Gdansk. Only the patients with all necessary measurements were included in the study. All groups included patients generally healthy or with diabetes or/and smokers. Patients with other systemic diseases and patients with dental implants were excluded. A dental assessment of the patients was performed, and the following indicators were included: gender, age, active nicotinism, the number of preserved teeth, approximal plaque index, bleeding on probing, pocket depth, and clinical attachment loss. The measurements were performed by one dentist with the use of a standardized periodontal probe with 15 mm scaling. The study only included participants with all required measurements. Stadium I periodontitis affected 12, stadium II periodontitis affected 19, stadium III periodontitis affected 42, stadium IV periodontitis affected 27, and gingivitis affected 10. Patients who were usually healthy, had diabetes, or smoked were included in all categories. Patients having dental implants and those with other systemic disorders were not included.

### 2.2. Network and Programming

#### 2.2.1. Basics of Kohonen Neural Networks

In 1982, in the article titled “Self-Organized Formation of Topologically Correct Feature Maps”, T. Kohonen proposed a new algorithm of artificial neural networks, which was named Kohonen networks [18]. Those networks can be characterized as self-learning with built-in competition and a neighborhood mechanism. They are constructed from two layers: input and output. Self-learning is based on the fact that learning, also known as network training, takes place in the “unsupervised learning” (self-organizing) mode, which means that for the given input data for training there is no presented correct answer.

The network is not familiar with what output signals should correspond to the input signals. Competition is the mechanism by which neurons learn to recognize input signals by competing with each other. The neuron which reacts most strongly to a given input signal wins: the more the neurons’ weights are similar to the input signals (input values), the stronger the reaction “wins” in the competition of recognizing specific input signals. Other neurons become winners in recognizing other input signals (values). Neighborhood is understood here as such teaching of the network that the neighbors of the neuron that are victorious in recognizing specific signals learn along with it, although less intensely. Such network training causes the neighboring neurons to respond to similar input signals (values). The training result of the network (output layer neurons) is plotted in a graph called a Kohonen map or topological map. The individual observations are called input or training cases.

#### 2.2.2. Architecture and Training

The KNN architecture consists of a multi-dimensional input layer and a typically one-dimensional or two-dimensional output layer. The neurons fight with one another in the output layer, also known as the competitive layer, and only one is chosen as the winner, or put another way, as the class most appropriate for a certain input vector x. Each component of the input vector is connected to every component of the output layer in these networks. Weight w_ij_ between the input neurons j and the output layer’s neurons i serves as a proxy for the strength of the connections.

The Euclidean distances D_i_ between the input vector and the weights connected to each of the output neurons are calculated during the training of the KNN model, as indicated by the following equation:

(1) $$D_{i}=\sqrt{\sum_{j=1}^{K}{\left(x_{j}-w_{\mathrm{ij}}\right)}^{2}},\;i=1,\;2,\;3,\;\ldots ,\;L,$$

where K is the input vector x’s dimension, L is the total number of neurons in the output layer, and x_j_ is the input vector x’s j-th component.

The winner neuron is the output neuron i with the least Euclidean distance relative to the input vector. The Kohonen rule [19] is then used to update the weights related to this neuron i and the neurons nearby V_i*_, as stated in the following equation:

(2) $$w_{\mathrm{ij}}\left(n\right)=w_{\mathrm{ij}}\left(n-1\right)+\propto [x_{j}\left(n\right)-w_{\mathrm{ij}}\left(n-1\right)],\;i\in V_{i^{*}\;},\;j=1,2,\;\ldots ,\;K$$

where n is an index that specifies the order in which samples are presented to the network, and α is the learning rate.

The Euclidean distance becomes lower as a result of the Kohonen rule, which drives the weights linked to the winner neuron and its neighbors to move in the direction of the input vector provided to the network. As a result, these neurons learn to identify related vectors. The full dataset can also be used to present input vectors to the network prior to any weight updates. Batch mode is the name given to this display style. In this scenario, each input vector is searched for the winning neuron, and the weight vector is then changed to a position determined by the average of the input vectors for which the winning neuron or its neighbor was present. After several iterations of the input dataset presentations, the weights typically stabilize.

#### 2.2.3. Application of the KNN Model

In our study, the structure of the Kohonen network was not complicated compared to other types of neural networks. The Kohonen network consists of input and output layers, but it does not have any hidden layers, as with other types of networks. Technical data for network maintenance have been standardized. The data were normalized before scheduling so that the average would be 0 and the unit standard deviation would be 1. Each patient was represented by a vector of the number of coordinates and factors that need to be taken into account: in our case, 171 variables initially, in order to generate a SOM (sex, age, smoking, oral hygiene, periodontal pocket depth, and maximum interproximal loss of connective tissue attachment); see Table 1. The patients were divided into blocks called neurons using an iterative method with the goal that the patients making up each neuron have similar characteristics and distinct ones from those making up other neurons.

In this study, the vectors of the input layer had 195 neurons representing information from the patients’ records regarding sex, age, smoking, oral hygiene, periodontal pocket depth, and maximum interproximal loss of connective tissue attachment (Table 1). Each patient was represented by a vector of the number of coordinates and factors that need to be taken into account, in our case, 171 variables initially, in order to generate a SOM.

A popular method of mapping elements into layers was divided into its forms of a two-dimensional network, and shared with (rectangles, circles) in preparation from the software that corresponds to individual neurons. 

At the start of the SOM, a decision must be made regarding the number of neurons and, consequently, the number of groups to form. Between a few dozen and several thousand neurons may exist. In our instance, the number of patients and variables to be researched led us to select a collection of 16 neurons which are related to the periodontitis grade (Healthy, A–C) and stage (I–IV). The distribution of the patients on the map after the SOM training process revealed that certain neurons had more patients than others or even had empty neurons. Not one of our patients had the pattern that corresponded to that neuron, as seen by empty neurons.

#### 2.2.4. Computer Processing and Program

We used a computer Intel^®^ Core™ i7–9850H CPU© 2.60 GHz, 16 GB RAM, and 512GB HDD. The algorithm described by Haykin [20] was applied to the neural network routine that was created, and Statistica Automated Neural Networks, TIBCO Software Inc. (2017). Statistica (data analysis software system), version 13. http://statistica.io (accessed on 13 January 2023) was used to process the results. 

## 3. Results

### 3.1. Basic Characteristics

Of the 110 patients, 65.5% were female, and the study group included 9.1% of healthy individuals free of periodontitis, 13.6% of patients with grade A, 39.1% of patients with grade B, and 38.2% of patients with grace C. In total, 10.9% of the study group’s patients had stage I periodontitis, followed by stage II patients 17.3%, stage III patients 38.2%, and stage IV patients 24.5%. The average age was 45.2 (95% CI: 43.8–46.6). The mean approximal plaque index (API) was 77.9% (95% CI: 73.4–82.4), bleeding on probing index (BOP) was 60.0% (95% CI: 53.5–66.5), interproximal clinical attachment loss (CAL) was 3.63 mm (95% CI: 3.19–4.08), and pocket depth was 2.90 mm (95% CI: 2.75–3.05). Most of the patients (about two-thirds) had non-physiologic tooth mobility. Over 68% of the study group had furcation that could not be detected.

### 3.2. SOM Analysis

**Figure 1:**
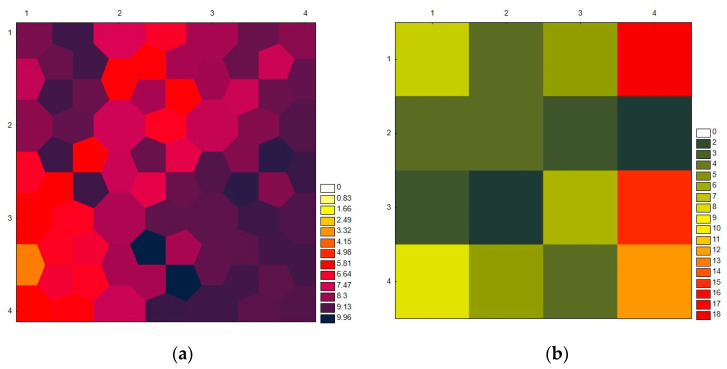
Distribution of SOM: (**a**) distances between neurons of a KNN model for the determination of the neighborhood; (**b**) distribution of the study participants among each neuron while accounting for the 171 study-related variables.

Each patient was represented by a vector made up of as many components and variables as there were in the study, or initially 171 variables, as described in the preceding section. When the patients had comparable traits, we could classify them into neurons by analyzing the minimal distances between those vectors. Our study used a map of 171 neurons because there were so many instances involved, and the 110 patients were dispersed among them as indicated in Figure 1a,b.

Using the criteria outlined in the Material and Methods section, where a higher percentage of a neuron’s filling denotes a greater number of patients with that pattern and an empty neuron denotes the absence of any cases exhibiting the characteristics associated with that neuron, we can see that neuron 13 had the most patients with 19 and that neurons 1, 4, 15, and 16 each had 8–16 patients. Between one and seven patients were present in the other neurons. As a result, each neuron displayed the many patient patterns that were discovered during our study (Figure 1b).

**Figure 2:**
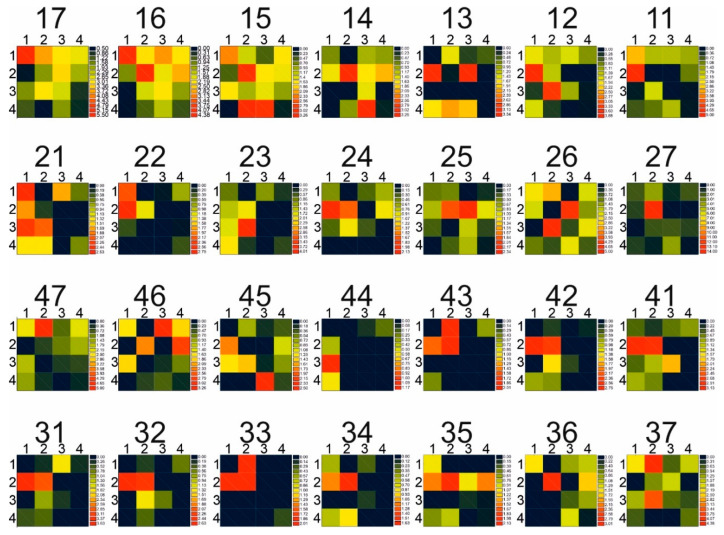
The distribution of study variable values in each neuron that contains a pattern of patients with a comparable minimum distance in accordance with the artificial neural network algorithm for the 28 variables (clinical attachment loss, all tooth) taken into consideration is referred to as the map component for each of the 16 variables. Over each map, the variable under analysis is shown.

The distribution of each of these magnitudes in the pattern corresponding to each neuron is shown in Figure 2 for the clinical attachment loss of the 28 variables included in the study on a color scale. Because the procedure projected the value of the variable that would correspond to that neuron, it can be seen in this situation that every neuron, including the empty ones, had a value assigned for the variable specified. The pattern associated with those vacant neurons is unimportant to our investigation because no patients were allocated to them during the course of it.

It is clear that some variables’ values greatly varied between the investigated neurons, whereas their values in other neurons were more or less the same (Table 2).

To discover the pattern of patients according to the periodontitis grade and stage, we grouped the neurons together to form three clusters: Group 1 was made up of neurons 12 and 16 that represented a percentage of slow progression of almost 75%; Group 2 was made up of neurons 3, 4, 6, 7, 11, and 14 in which the percentage of moderate progression was almost 65%; and Group 3 was made up of neurons 1, 2, 5, 8, 9, 10, 13, and 15 that represented a percentage of rapid progression of almost 60%. Table 3 displays the pattern for each of the groups taken into consideration, and a variance analysis revealed the variables that were important for differentiating between these three patient groups. The significance values for each variable are shown in Table 3. There were no statistically significant differences in age, sex, and smoking on periodontitis progression. Mean values for API in the slow, moderate, and rapid progression groups were 59.71 vs. 82.27 vs. 89.56. There were statistically significant differences in API, BoP, PD, CAL, mobility, and furcation versus groups (*p* < 0.0001). The post-hoc tests showed that API values were significantly lower in Group 1 relative to Group 2 (*p* = 0.0010) and Group 3 (*p* = 0.0001). Furthermore, the BoP was significantly lower in Group 1 relative to Group 2 (*p* = 0.0029) and Group 3 (*p* = 0.0002). A detailed statistical analysis of PD showed that the PD value was significantly lower in Group 1 relative to Group 2 (*p* = 0.0001). Furthermore, the PD was significantly higher in Group 3 relative to Group 2 (*p* = 0.0068). There was a statistically significant CAL difference between Group 1 relative to Group 2 (*p* = 0.0370). The post-hoc tests showed that mobility was significantly lower in Group 1 relative to Group 2 (*p* = 0.0121) and Group 3 (*p* = 0.0002). A detailed statistical analysis of furcation showed that the furcation was significantly lower in Group 1 relative to Group 2 (*p* = 0.0057). Furthermore, the furcation was significantly higher in Group 3 relative to Group 2 (*p* = 0.0015) (Table 3).

## 4. Discussion

Nowadays, the disciplines of medicine and dentistry are becoming more and more dependent on artificial intelligence (AI) [15,21]. It can be helpful in a range of circumstances when new technology could be advantageous and helpful to people. In the medical field, AI can be useful, especially in fields such as radiology, pathomorphology, oncology, cardiology, psychiatry, nuclear medicine, and many others [22,23,24,25,26]. The use of computer models of neural networks is one way to understand how the nervous system functions, which we are unable to study under natural conditions due to the limitations of current research techniques [27,28,29,30,31]. Recent years have seen a significant increase in the application of in silico approaches to find novel pharmaceutical treatments for conditions such as cancer, autoimmune disease, and neurodegeneration [32,33,34].

Periodontitis progression evaluation is a crucial phase in the treatment planning process for a dentist, and it may also be useful in encouraging patients to actively engage in their care. The study mentioned above also considers periodontitis staging and grading according to the classification from 2017 of periodontitis. The relationship between plaque (API) and the onset of periodontitis is widely understood. Gingivitis will occur in all individuals who do not brush their teeth properly; however, the development of periodontitis is more complicated, more varied, and depends on numerous circumstances. The immune-inflammatory response of the host, which is dependent on genetic polymorphism, must participate [35,36]. The development of a disease is determined by these polymorphisms as well as environmental factors [37,38,39]. The primary indicator of gingivitis and periodontitis is bleeding on probing (BoP), which also distinguishes between localized and generalized forms of each ailment and provides information on the degree of inflammation. Additionally, a significant percentage of pockets with a depth of more than 6 mm signal greater severity. There are already some studies in which radiographic bone loss is measured with the use of artificial neural networks. To improve the quality and efficiency, deep learning models with the use of panoramic radiographs or intraoral radiographs have been developed to assist clinicians in interpreting and measuring alveolar bone to reach a periodontal diagnosis with high accuracy and reliability [40,41]. Kohonen networks are used in other medical fields, for example, in detecting breast cancer. In the study of Ashokkumar et al. deep learning techniques have been proposed as a potential way to accurately predict breast cancer in its early stages. The Kohonen self-organizing algorithms, feed forward, and radial basis functions are examples of assessment techniques for artificial neural networks. The outcomes of the study indicate that the deep learning model can more accurately assess the final diagnosis of the axillary lymph node metastatic from US imaging of initial breast cancer [42]. Kohonen’s artificial neural networks were also used to select new inhibitors of SARS-CoV-2 activator protein furin. In this research, it was found that 15 existing FDA antiviral drugs can have the potential to inhibit furin. Kohonen’s self-organizing maps (SOM) are widely employed today in pharmaceutical research to establish the connection between structure and biological activity for medication discovery [43]. In the study of Zhao Y et al. an upgraded collaborative neural network model was suggested in order to address the self-organizing mapping network’s Kohonen layer structure. The study investigated the relationship between branch retinal vein occlusion and arteriosclerosis by quantitatively measuring retinal vessel diameter and choroidal thickness with the use of Kohonen networks [44]. In addition, in psychiatry, neural networks can be successfully used, for example, in the study of Loula et al. where according to Brazilian statistics on mortality and the prevalence of major depressive disorder, a virtual population was created, and its five different types of inhabitants were clustered using Kohonen’s self-organizing map (SOM) [45]. In another study, Kohonen networks were used to assess the nutrition quality with frailty syndrome among the elderly [46]. Self-organizing maps (SOMs) were used with the socio-demographic data such as age, gender, and race to perform the correct classification of asthma outcome. Kohonen self-organizing maps, especially when integrating multi-dimensional data, are effective classification models for studying asthma outcomes, according to the study’s findings [47]. In the dermatologic study of Styła et al. the dermatoscopic images were used to train Kohonen neural networks to provide fully automatic diagnostic systems capable of determining the type of pigmented skin lesion [48]. Referring to the above and recent studies, it was shown that machine learning algorithms, particularly Kohonen networks, might be useful in medicine and can improve diagnosis and give clinicians more tools in treatment planning [42,43,44,45,46,47,48,49,50,51,52]. According to our study, we can recommend other specialists use Kohonen networks in their daily practice to ease the prediction of the progression of periodontitis with the usage of data: gender, age, active nicotinism, the number of teeth still present, the approximate plaque index (API), bleeding on probing (BoP), pocket depth (PD), and clinical attachment loss. After giving all of these input data, neural networks may predict the possibility of the progression of periodontitis that may be helpful for the clinicians, researchers, but also for the patients to outline the severity and probability of progression of the disease. 

The methodology that we have employed allows us to notice some of variables which present statistically significant differences in terms of the probability of progression. The dependences of these magnitudes do not appear when a customary statistics method comparing the means between the several groups is undertaken [21].

## 5. Conclusions

We discovered the pattern of patients according to the periodontitis grade and stage, and grouped the neurons together to form three clusters: Group 1 represented a percentage of slow progression of almost 75%; Group 2 in which the percentage of moderate progression was almost 65%; and Group 3 represented a percentage of rapid progression of almost 60%. More map nodes are shared between patients from Groups 2 and 3 than the more narrowly focused Group 1. When examining the patterns of each of these groups, it becomes clear that Groups 2 and 3 are interconnected, since we identify neurons that contain examples from both of these groups. However, this circumstance is a reflection of reality, rather than a flaw in the network.

To conclude, we can say that self-organizing maps can be taken into consideration while assessing the risk of the progression of periodontitis. It can be helpful especially for clinicians, but also for scientists while defining the stage of periodontitis.

## Figures and Tables

**Table 1:** Variables represented on the SOM.

Variable	Description	Valuation
Sex	Woman/man	0 = woman. 1 = man
Age	Initial age on beginning treatment	Decimal age (years)
Smoking	Smoking cigarettes	0 = no. 1 = yes
API ^1^	Approximal plaque index	0 = no. 1 = yes
BoP ^1^	Bleeding on probing	0 = no. 1 = yes
PD ^1^	Pocket depth	Decimal (mm)
CAL ^1^	Clinical attachment loss	Decimal (mm)
Mobility ^1^	Tooth mobility	0 = normal (physiologic) tooth mobility; 1 = detectable mobility (up to 1 mm horizontally); 2 = detectable mobility (more than 1 mm horizontally); 3 = detectable vertical tooth mobility
Furcation ^1^	Severity of furcation involvement	0 = furcation not detectable; 1 = furcation detectable, with a horizontal component of probing ≤3 mm; 2 = furcation detectable, with a horizontal component of probing >3 mm; 3 = furcation is opened through and through

^1^ for each tooth (28).

**Table 2:** Average values of the patient pattern corresponding to each neuron (N) considered.

Neuron	Sex	Age	Smoking	API	BoP	PD	CAL	Mobility	Furcation
1	0.3	46.5	0.3	0.91	0.93	4.26	6.12	0.65	0.17
2	0.8	42.3	0.5	1.00	0.89	3.34	4.37	0.37	0.09
3	0.5	47.0	0.2	0.94	1.00	3.02	4.59	0.20	0.03
4	0.4	43.9	0.1	0.91	0.96	2.91	3.75	0.09	0.02
5	0.5	45.5	0.8	0.87	1.00	3.26	4.04	0.42	0.08
6	0.3	46.3	0.3	0.73	0.76	3.26	3.79	0.39	0.07
7	0.7	47.0	0.0	0.79	0.67	2.75	4.56	0.30	0.01
8	1.0	39.0	0.5	0.68	0.76	2.99	2.48	0.02	0.01
9	0.3	52.3	0.3	0.86	1.00	2.76	3.21	0.35	0.02
10	0.0	46.0	0.5	0.66	0.38	3.32	4.56	0.28	0.02
11	0.0	50.3	0.3	0.69	0.44	2.68	2.95	0.21	0.01
12	0.3	42.2	0.1	0.54	0.41	2.40	2.88	0.01	0.01
13	0.6	52.0	0.3	0.97	0.32	3.29	3.21	0.34	0.10
14	0.0	51.7	0.3	0.65	0.38	2.59	3.17	0.35	0.01
15	0.0	43.8	0.0	0.78	0.46	2.96	3.91	0.02	0.01
16	0.4	38.3	0.3	0.57	0.20	2.18	1.92	0.03	0.01

**Table 3:** Periodontitis grade: probability of progression.

	Group 1	Group 2	Group 3	*p*-Value
Progression	Slow	Moderate	Rapid	
Sex	0.3	0.3	0.4	0.4827
Age	43.4	47.3	47.0	0.0638
Smoking	0.2	0.2	0.4	0.1028
API	0.60	0.82	0.90	<0.0001
BoP	0.34	0.65	0.74	<0.0001
PD	2.40	2.90	3.41	<0.0001
CAL	2.51	3.72	4.19	0.0212
Mobility	0.02	0.21	0.37	<0.0001
Furcation	0.02	0.04	0.16	0.0007

## Data Availability

All data generated or analyzed during this study are included in this published article.
